# Identification and Phylodynamic Analysis of the Siberian Subtype of Tick‐Borne Encephalitis Virus in Tick‐Bitten Patients From Northeastern China

**DOI:** 10.1155/tbed/3756652

**Published:** 2025-12-11

**Authors:** Zhiwei Wei, Ziyan Liu, Yu Liu, Ning Liu, Yuanning Ren, Shuzhen Han, Xiangyu Zheng, Xiaolong Lv, Zedong Wang

**Affiliations:** ^1^ Department of Infectious Diseases, Center of Infectious Diseases and Pathogen Biology, State Key Laboratory of Zoonotic Diseases, The First Hospital of Jilin University, Changchun, Jilin Province, China, jlu.edu.cn; ^2^ Department of Neurology and Neuroscience Center, The First Hospital of Jilin University, Changchun, Jilin Province, China, jlu.edu.cn; ^3^ Department of Neurology, The Second Affiliated Hospital of Inner Mongolia University for the Nationalities, Inner Mongolia Forestry General Hospital, Yakeshi, Inner Mongolia Autonomous Region, China, imun.edu.cn; ^4^ International Center of Future Science, Jilin University, Changchun, Jilin Province, China, jlu.edu.cn

**Keywords:** Northeastern China, Siberian subtype, tick-bitten patients, tick-borne encephalitis virus (TBEV), ticks

## Abstract

Far Eastern (FE) tick‐borne encephalitis virus (TBEV) was thought to be the only endemic subtype in northeastern China. However, in 2024, the Siberian (Sib) subtype of TBEV was found in ticks. This study investigates Sib‐TBEV infection in tick‐bitten patients in the region. A total of 2573 tick‐bitten patients, including 506 inpatients and 2067 outpatients, were enrolled between April and September in both 2023 and 2024 and were tested for Sib‐TBEV using real‐time quantitative polymerase chain reaction (RT‐qPCR). Seven cases were confirmed, with four among inpatients and three among outpatients. All four inpatients exhibited headaches and had hospital stays ranging from 5 to 21 days. Two inpatients with longer hospital stays showed additional symptoms: one experienced dizziness and fatigue, while the other exhibited liver injury and neurological symptoms, including facial paralysis, dysarthria, and meningeal signs. All patients recovered and were discharged without sequelae. Seven Sib‐TBEV complete genomes were amplified and classified into the Sib‐TBEV Zausaev and Vasilchenko lineages. Bayesian analysis estimated that the most recent common ancestor (tMRCA) of these strains dates to a period between 1898 and 1983, with possible migration pathways from Irkutsk or Zabaykalsky to the Daxing’an Mountains (DXAM) in northeastern China. We identified Sib‐TBEV‐infected patients in northeastern China and characterized their epidemiological and clinical features. Sib‐TBEV may have been circulating in northeastern China for decades, underscoring the need for TBEV surveillance in ticks, animals, and tick‐bitten patients.

## 1. Introduction

Tick‐borne encephalitis virus (TBEV), a member of the genus *Orthoflavivirus* in the family *Flaviviridae*, is one of the most important arboviral pathogens affecting the human central nervous system in Eurasia [1, 2]. TBEV has traditionally been classified into three subtypes: European (Eu)‐TBEV, Siberian (Sib)‐TBEV, and Far Eastern (FE)‐TBEV [3]. The geographical distribution, clinical symptoms, and pathogenicity of Eu‐, Sib‐, and FE‐TBEV differ from each other. Eu‐TBEV, prevalent in European countries, causes milder symptoms and has the lowest case fatality rate, ranging from 0.5% to 2%. Sib‐TBEV, widely distributed in Siberian Russia, Eastern Europe, and Central Asia, is associated with severe symptoms and chronic infection, with a case fatality rate of 6% – 8%. FE‐TBEV, endemic to FE Russia and parts of Northeast Asia, can lead to severe neurological manifestations and has a case fatality rate of 20%–40% [4]. In recent years, two additional TBEV subtypes have been identified: Baikalian (Bkl)‐TBEV, which originated from Sib‐TBEV, and Himalayan (Him)‐TBEV, discovered in *Marmota himalayana* in northwestern China [5, 6]. These findings highlight the genetic diversity of TBEV and underscore its growing public health significance.

In China, TBEV is primarily disseminated in the forested regions of the northeastern part, with ~90% of cases being reported [7]. FE‐TBEV is considered to be the sole endemic subtype in northeastern China, although Sib‐TBEV has been detected in *Ixodes persulcatus* ticks for years from areas of Russia (Zabaykalsky Krai) adjacent to northeastern China [8, 9]. However, a recent 2024 study reported the first identification of Sib‐TBEV in the Daxing’an Mountains (DXAM) region of China, revealing the potential for complex prevalence involving both FE‐ and Sib‐TBEV subtypes in this area [10].

In this study, we conducted active surveillance for Sib‐TBEV in tick‐bitten patients in the DXAM using real‐time quantitative polymerase chain reaction (RT‐qPCR), analyzed their epidemiological, clinical, and laboratory characteristics, and performed phylogenetic and phylodynamic analyses on the newly identified Sib‐TBEV strains from these patients. The findings will provide new insights into the epidemic trends and comprehensive prevention and control of TBEV in China.

## 2. Materials and Methods

### 2.1. Study Design and Sample Collection

In this active surveillance study, we conducted monitoring at the General Forestry Hospital of Inner Mongolia, the largest tick‐borne disease sentinel hospital in the DXAM region of Northeast China, and a center recognized for its expertise in treating tick‐borne encephalitis. Individuals who sought medical attention due to a recent tick bite between April 1 and September 1, 2023–2024, were enrolled in the study. Sera samples were collected on admission and stored at −80°C until use. Demographic details, medical history, and tick bite information were collected via a standardized survey. Two authors extracted and cross‐verified clinical symptoms, pre‐existing conditions, laboratory findings, treatment methods, and patient outcomes from the medical records.

### 2.2. Laboratory Confirmation of TBE Cases

TBEV IgG and IgM antibodies were detected using the TBE virus IgG/IgM IIFT kit (EUROIMMUN, Germany). Total RNA was extracted from each participant’s serum using the MagaBio Plus Virus DNA/RNA Purification Kit III (BIOER, China) and then reverse‐transcribed into cDNA with the PrimeScript 1st Strand cDNA Synthesis Kit (TaKaRa, Japan), following the manufacturer’s instructions. Sib‐TBEV‐specific RNA was detected by RT‐qPCR using a primer pair (Forward: 5′‐GGTGGTKTCYCTYTTYAC‐3′, Reverse: 5′‐CCYCCAAGGTCTCTCATG‐3′) and a probe (5′‐FAM‐TAYATCATCCACCARCTBCAGACCAA‐BHQ1‐3′) designed based on Sib‐TBEV strains retrieved from the NCBI GenBank database. Amplification was carried out using Premix Ex Taq (qProbe PCR) (TaKaRa, Japan) under conditions specified by the manufacturer. A Ct value ≤35 was considered positive. The complete genome sequences of Sib‐TBEV‐positive samples were amplified using overlapping semi‐nested PCR with specific primers (Supporting Information 1: Table S1) and sequenced by the Sanger method.

In addition to Sib‐TBEV, we tested for numerous other tick‐borne pathogens prevalent in the DXAM region, including Alongshan virus, Yezo virus, Beiji nairovirus, Songling virus, Wetland virus, FE‐TBEV, *Borrelia* spp., *Rickettsia* spp., *Anaplasma* spp., and *Babesia* spp., using the methods and primers listed in Supporting Information 1: Table S2.

To prevent cross‐contamination and ensure accuracy, we followed these measures: (i) separate pre‐ and post‐PCR areas; (ii) clean surfaces and equipment with 1% (vol/vol) sodium hypochlorite; (iii) use UV irradiation for decontamination; (iv) include negative controls to monitor contamination; and (v) confirm results by semi‐nested PCR amplification.

### 2.3. Phylogenetic and Homology Analysis

Phylogenetic analysis was performed using the maximum likelihood method with the JTT model in MEGA 7.0, utilizing Sib‐TBEV strains identified in this study and reference sequences obtained from GenBank (Supporting Information 1: Table S3). Sequence similarity was evaluated using the MegAlign tool within the DNAstar software package, and the results were visualized via a heatmap generated with GraphPad Prism 8.

### 2.4. Bayesian Phylodynamics Analysis

To elucidate the origin of Sib‐TBEV strains in northeastern China, Bayesian analysis was conducted to estimate the time of the most recent common ancestor (tMRCA) of the viruses. Given that the E protein of Sib‐TBEV has a significantly larger number of available sequences compared to whole‐genome data, and considering that phylogenetic resolution was not substantially compromised when using E protein sequences relative to full genome sequences, we adopted the approach used in previous studies and selected the E protein as the target gene for analysis [6, 11, 12]. A total of 178 E protein nucleotide sequences of Sib‐TBEV, including seven strains identified in this study, were included in the phylodynamic analysis (Supporting Information 1: Table S4). As previously described, the analysis was conducted using the BEAST 1.10.4 software package, with the optimal nucleotide substitution models—GTR + G4 and HKY + G4—selected based on evaluation by IQ‐TREE 2.1.3 [13, 14]. Convergence was checked in Tracer v1.7, requiring ESS > 200 [15]. The maximum clade credibility (MCC) tree was generated with TreeAnnotator and visualized using FigTree v1.4.3 [16]. Bayes factor (BF) and posterior probability (PP) values were calculated using SpreaD3, and the primary migration pathways were inferred based on BF > 3 and PP > 0.5 [17].

## 3. Results

### 3.1. Epidemiological and Clinical Features

**Figure 1:**
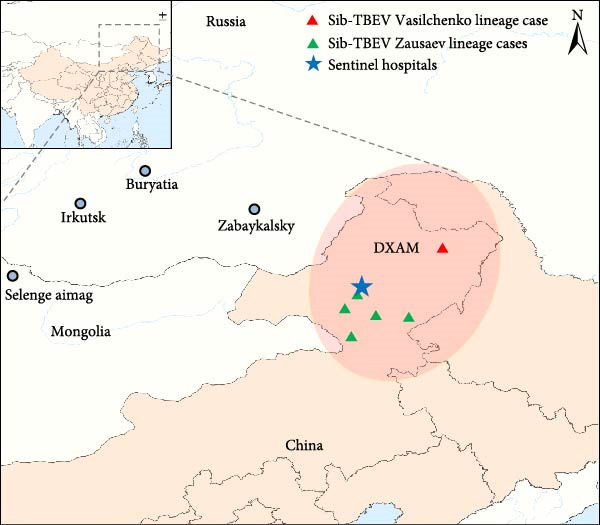
Geographical distribution of patients with Sib‐TBEV infection in northeastern China. The TBEV natural foci in the DXAM of northeastern China is indicated by red oval. Green and red triangles represent cases of Sib‐TBEV Zausaev and Vasilchenko lineage infections, respectively. Blue star marks the location of the sentinel hospital.

Using RT‐qPCR, we successfully confirmed seven Sib‐TBEV‐positive patients in DXAM (Figure 1). Of these, four were hospitalized, while the other three were outpatients (Table 1). The seven patients, aged 26–53 and including five males, were tick‐bitten between May and June, with incubation periods ranging from 7 to 28 days (Table 1, Supporting Information 1: Table S5, Figure S1). All developed fever, with higher peak temperatures observed in inpatients than in outpatients. None had a history of TBEV vaccination. All four inpatients exhibited headaches and had hospital stays ranging from 5 to 21 days. The two inpatients with longer hospital stay showed additional symptoms: one experienced dizziness and fatigue, while the other exhibited liver injury and neurological symptoms, including facial paralysis, dysarthria, and meningeal signs (Table 1). None of the four patients required admission to the intensive care unit, and all achieved a favorable prognosis (Table 1).

**Table 1:** Epidemiological characteristics and clinical symptoms of seven Siberian subtype tick‐borne encephalitis virus‐infected patients.

Items	HLB‐ZH148	HLB‐ZG50	HLB‐ZH141	HLB‐H168	HLB‐H197	HLB‐H372	ARS‐H457
Epidemiological characteristics							
Age (years)	26	35	47	53	38	34	42
Sex	Male	Female	Female	Male	Male	Male	Male
TBEV vaccination	No	No	No	No	No	No	No
Year of collection	2024	2023	2024	2024	2024	2024	2024
Tick bite month	June	June	June	May	June	June	May
Incubation period (days)	12	10	10	11	7	4	24
Hospitalization	Yes	Yes	Yes	Yes	No	No	No
Hospital stay duration (days)	21	5	10	17	NA	NA	NA
Clinical manifestations and signs							
Fever	Yes	Yes	Yes	Yes	Yes	Yes	Yes
Highest temperature (°C)	40.0	39.0	39.1	38.9	37.8	37.9	38.0
Headache	Yes	Yes	Yes	Yes	Yo	Yo	Yo
Dizziness	Yes	No	No	No	No	No	No
Fatigue	Yes	No	No	No	No	N o	No
Facial paralysis	No	No	No	Yes	No	No	No
Dysarthria	No	No	No	Yes	No	No	No
Meningeal signs	No	No	No	Yes	No	No	No
Liver injury	No	No	No	Yes	No	No	No
Clinical severity							
ICU care	No	No	No	No	No	No	No
Death	No	No	No	No	No	No	No

Abbreviation: NA, not available.

### 3.2. Phylogenetic and Homology Analysis

The complete genome sequences of all seven cases were successfully amplified (Supporting Information 1: Table S6). Phylogenetic analysis grouped the six strains (HLB‐ZG50, HLB‐ZH141, HLB‐H168, HLB‐H197, HLB‐H372, and ARS‐H457) and two additional tick‐derived strains (HL10 and HL11) from northeastern China into the Sib‐TBEV Zausaev lineage. The remaining strain, HLB‐ZH148, together with HL9 (tick origin), clustered into the Sib‐TBEV Vasilchenko lineage (Figure 2A). The Sib‐TBEV strains identified in northeastern China exhibited high sequence identities with Russian Zausaev and Vasilchenko lineage strains, with nucleotide identities ranging from 93.9% to 99.0% and amino acid identities from 97.3% to 100.0% (Figure 2B, supporting data).

Figure 2Phylogenetic and homology analysis of the identified Sib‐TBEV in northeastern China. (A) Phylogenetic tree based on the polyprotein amino acid sequences of Sib‐TBEV. (B) Nucleotide (lower left) and amino acid (upper right) sequence identities of Sib‐TBEV polyprotein. The tree was constructed by the maximum likelihood method with the JTT model using MEGA 7.0. Red font indicates strains identified in this study, while purple font denotes Sib‐TBEV strains identified in ticks in northeastern China.

**Figure:**
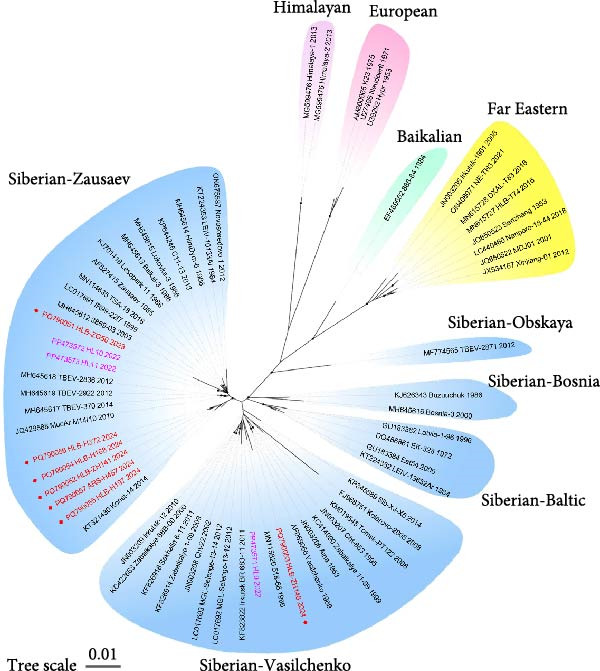
(A)

**Figure:**
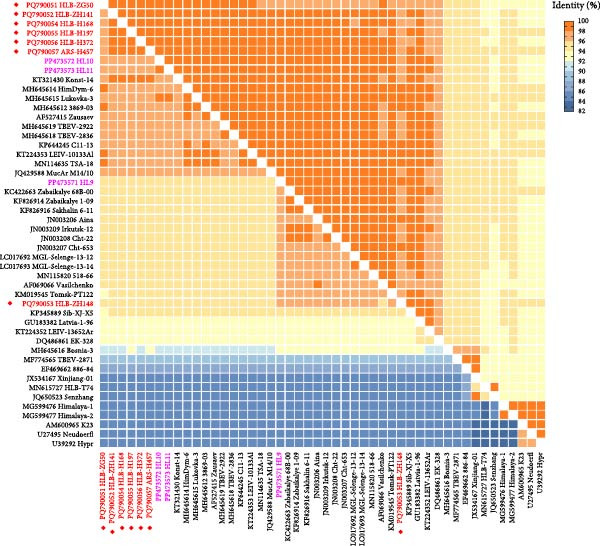
(B)

### 3.3. Bayesian Phylodynamic Analysis

Bayesian phylogenetic analysis revealed that the Sib‐TBEV Zausaev lineage strains identified in northeastern China were estimated to have a median tMRCA ranging from 1898 to 1983, while the Sib‐TBEV Vasilchenko lineage strains were estimated to have a median tMRCA ranging from 1946 to 1977 (Figure 3A). The migration pathways for the Sib‐TBEV Zausaev lineage to northeastern China were identified as follows: from Western Siberia to Irkutsk, and then to DXAM. For the Sib‐TBEV Vasilchenko lineage, the pathways included routes from Western Siberia or Irkutsk to Buryatia, or from Irkutsk or Buryatia to Zabaykalsky, ultimately reaching DXAM (Figure 3B). The areas exporting and importing Sib‐TBEV are presented in a histogram (Figure 3C). Zabaykalsky and Irkutsk in Russia are the primary sources of Sib‐TBEV export, while a few export incidents have been predicted in DXAM in northeastern China.

Figure 3Bayesian phylodynamic analysis of the identified Sib‐TBEV in northeastern China. (A) Time‐scaled Bayesian MCC phylogenetic tree based on the nucleotide sequences of Sib‐TBEV E protein. The estimated median dates of time to the most recent common ancestor (tMRCA) and 95% highest posterior density were represent with red font. (B) Predicted origin and migration routes of Sib‐TBEV identified in northeastern China. Significant epidemiological unidirectional pathways from one location to another are marked on the map. Red arrows, very strongly supported rates with BF＞100; blue arrows, strongly supported rates with 10 ≤ BF < 100; and yellow arrows, supported rates with 3 ≤ BF < 10. The solid lines represent the migration routes of Siberian‐Vasilchenko, while the dotted lines represent those of Siberian‐Zausaev. The orange‐shaded area stands for the DXAM in China. (C) Histogram of total number of state transitions for the major sites.

**Figure:**
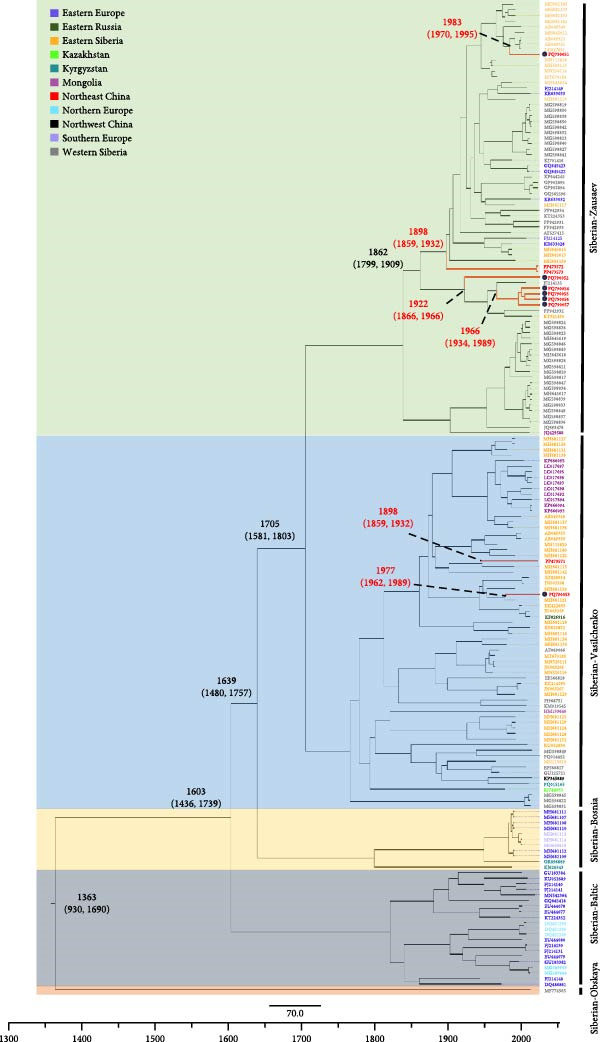
(A)

**Figure:**
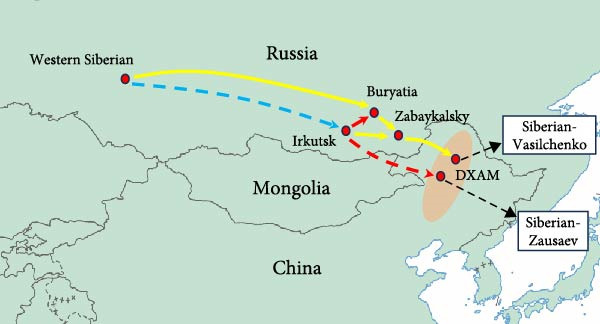
(B)

**Figure:**
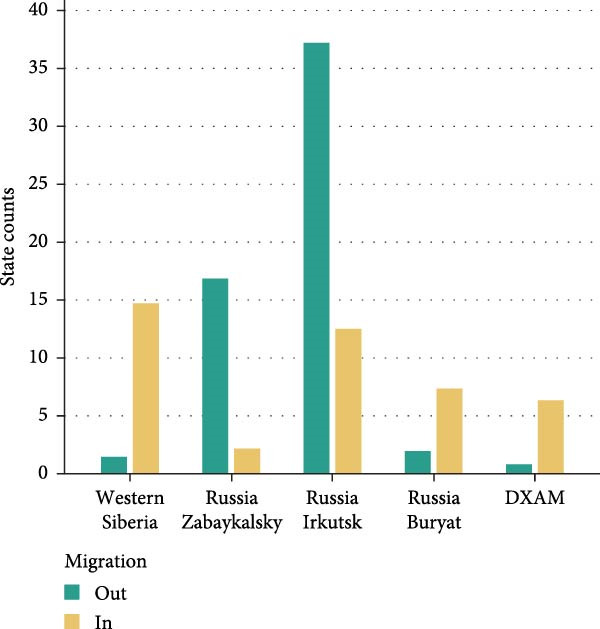
(C)

## 4. Discussion

In this study, seven tick‐bitten patients infected with Sib‐TBEV were identified in the DXAM of northeastern China for the first time, confirming findings from previous epidemiological studies on the presence of Sib‐TBEV in the DXAM ticks and indicating that Sib‐TBEV has become widely prevalent in this region. Although Sib‐TBEV can cause severe clinical manifestations and has a case fatality rate of 6%–8% [4], no severe or fatal cases were observed among the infected patients in this study. This may be due to the limited number of cases identified or the inability of serological testing alone to distinguish between FE‐ and Sib‐TBEV infections in severe cases after the viremic phase.

In China, the only vaccine against TBE was developed based on the inactivated Senzhang strain (FE‐TBEV subtype) and has been widely used since the 1970s [18]. In this study, none of the seven identified cases had been vaccinated with this vaccine, which helps eliminate the possibility that the clinical symptoms of these patients were alleviated as a result of vaccination.

In addition to Sib‐TBEV infections, we identified three FE‐TBEV‐positive serum samples from other tick‐bitten individuals, albeit at low viral copy numbers (data not shown). However, this finding does not indicate that Sib‐TBEV has become the dominant subtype in the region. Due to the shorter viremic phase of FE‐TBEV compared to Sib‐TBEV, viral nucleic acids are rarely detectable in clinical samples. Moreover, serological cross‐reactivity limits the ability to differentiate infections caused by the two subtypes using serological assays.

Sib‐TBEV contains at least five distinct lineages: Zausaev, Vasilchenko, Baltic, Obskaya, and Bosnia (Figure 2A) [8]. The Zausaev lineage, prevalent from Eastern Europe to Eastern Siberia, is the most commonly observed. In contrast, the Vasilchenko lineage is distributed across Western Siberia to the Far East region. The other three lineages are restricted to areas away from the Far East [8]. Our finding of six Zausaev strains further extended the distribution of this lineage from Eastern Siberia to the Far East.

Notably, our Bayesian phylogenetic analysis revealed the identified strains in northeastern China did not emerge recently and can be traced back to the late 19th century, indicating that Sib‐TBEV might have been present in China for decades (Figure 3A). However, Sib‐TBEV in the DXAM of northeastern China was not confirmed until 2024 [10]. The following factors may explain the prolonged underrecognition of Sib‐TBEV in northeastern China. Serological methods are commonly used in clinical settings to confirm TBEV infection, which limits the detection of Sib‐TBEV in tick‐bitten patients. Moreover, PCR detection efforts targeting TBEV in ticks in the DXAM region have primarily relied on FE‐TBEV‐specific sequences for primer design [9]. Metagenomic analyses of ticks have only been conducted in the past decade, and sampling sites have not adequately covered the current Sib‐TBEV endemic areas [19].

Although no Sib‐TBEV infections or infected ticks have been reported in other regions of Northeast China outside the DXAM, such as the Changbai Mountains [19–21], the combined effects of sporadic transmission events—particularly those driven by animal migration, including migratory birds—and factors such as climate change may ultimately lead to further expansion of the geographical distribution of Sib‐TBEV. The emergence of Sib‐TBEV poses new challenges for TBEV surveillance, prevention, and control in China, necessitating enhanced monitoring of Sib‐TBEV in ticks, animals, and tick‐bitten patients.

This study has several limitations. First, there is no effective serological method to differentiate between FE‐ and Sib‐TBEV positive patients, which may result in the number of identified cases being lower than the actual number of infections. Moreover, due to the limited number of Sib‐TBEV strains obtained in northeastern China, our Bayesian analysis results may deviate from objective facts.

## 5. Conclusions

We identified seven Sib‐TBEV‐infected tick‐bitten patients in DXAM of northeastern China for the first time, and characterized the epidemiological and clinical features of these patients. We also estimated the possible years and migration pathways that Sib‐TBEV transmit to northeastern China. Further efforts are needed to assess the geographical distribution and host range of this subtype in the region.

## Ethics Statement

This study was reviewed and approved by the Ethics Committees of the Inner Mongolia Forestry General Hospital (Approval Number NLZ‐2023‐013). Written informed consent was obtained from all participants or their legal guardians.

## Conflicts of Interest

The authors declare no conflicts of interest.

## Author Contributions

Zhiwei Wei, Ziyan Liu, and Yu Liu contributed equally to this work.

## Funding

This work was supported by the National Key Research and Development Program of China (2022YFC2601900 to Zedong Wang), the Science and Technology Program of the Joint Fund of Scientific Research for the Public Hospitals of Inner Mongolia Academy of Medical Sciences, China (2023GLLH0342 to Xiaolong Lv, and 2023GLLH0335 to Shuzhen Han).

## Supporting Information

Additional supporting information can be found online in the Supporting Information section.

## Supporting information


**Supporting Information 1** Figure S1: Timeline of seven patients with Sib‐TBEV infection. Table S1: Primers used for tick‐borne encephalitis virus detection and genome amplification. Table S2: The primers used to detect tick‐borne pathogens prevalent in the Daxing’an Mountains. Table S3: Reference tick‐borne encephalitis virus genome sequences utilized for phylogenetic analysis. Table S4: Reference Sib‐TBEV sequences utilized for Bayesian phylodynamic analysis. Table S5: Dates of clinical milestones and laboratory detection results of collected samples from seven patients infected with the Sib‐TBEV. Table S6: The information of the seven Sib‐TBEV strains obtained from the patients in northeastern China.

## Data Availability

The data that support the findings of this study are available from the corresponding author upon reasonable request.
