# Supplementary material for: Identification and Phylodynamic Analysis of the Siberian Subtype of Tick‐Borne Encephalitis Virus in Tick‐Bitten Patients From Northeastern China
Source: Transbound Emerg Dis. 2025 Dec 11;2025:3756652. doi: 10.1155/tbed/3756652 (PMC12698504; doi:10.1155/tbed/3756652)
Supplement: Supplementary file 1 — Supporting Information 1 Figure S1: Timeline of seven patients with Sib‐TBEV infection. Table S1: Primers used for tick‐borne encephalitis virus detection and genome amplification. Table S2: The primers used to detect tick‐borne pathogens prevalent in the Daxing’an Mountains. Table S3: Reference tick‐borne encephalitis virus genome sequences utilized for phylogenetic analysis. Table S4: Reference Sib‐TBEV sequences utilized for Bayesian phylodynamic analysis. Table S5: Dates of clinical milestones and laboratory detection results of collected samples from seven patients infected with the Sib‐TBEV. Table S6: The information of the seven Sib‐TBEV strains obtained from the patients in northeastern China. [file TBED-2025-3756652-s001.docx]

**Supplementary materials**

**
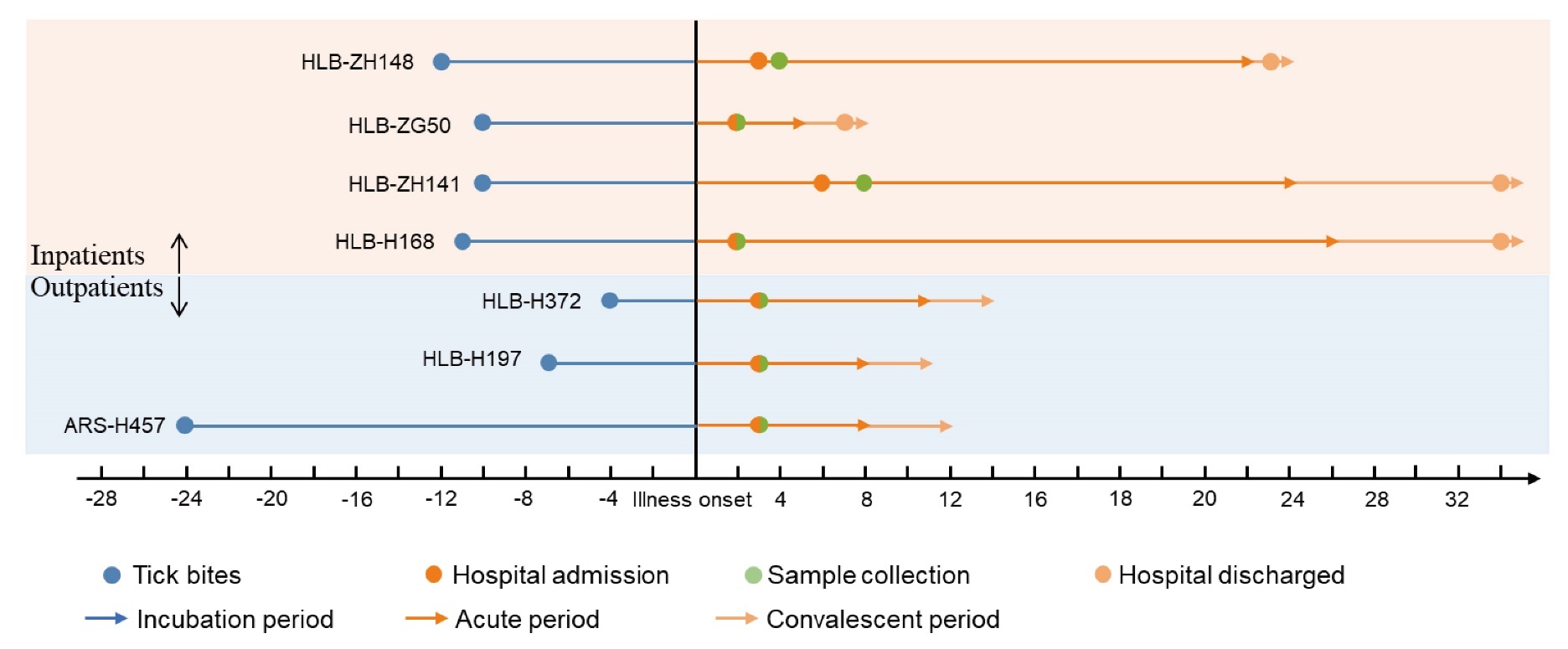
**

**Figure S1.** **Timeline of seven patients with Sib-TBEV infection.** The acute phase is defined by the onset of headache and fever. Colored arrows indicate disease phases, and distinct symbols mark key clinical milestones. Inpatients and outpatients are represented with orange and blue backgrounds, respectively.

**Table S1. Primers used for tick-borne encephalitis virus detection and genome amplification.**

| **Primer** |  | **Position (bp)** | **Sequence (5'→3')** | **Polarity** | **Amplicon (bp)** |
| --- | --- | --- | --- | --- | --- |
| **Detection** |  |  |  |  |  |
| TBEV-F |  | 11009 | GGGCGGTTCTTGTTCTCC | + | 67 |
| TBEV-R |  | 11053 | MCWCATCACCTCCTTGTCAGACT | - |  |
| TBEV-P |  | 11027 | AGCACAACAATCAACTCACTAAGCCT | + |  |
| **Genome amplification** | | | | | |
| 1 TBEV F |  | 1 | TTGCRTGCRTTTGCTTCGGAT | + | 398 |
| 1 TBEV R2 |  | 378 | TTCCACGTCTCTGYAAGCCTA | - |  |
| 1 TBEV R1 |  | 437 | AACACRAGYCCATTTGGCATT | - |  |
|  |  |  |  |  |  |
| 2 TBEV F |  | 324 | TGAAACAGGCCACGRCAGCA | + | 851 |
| 2 TBEV R2 |  | 1154 | CACTCTGGTCTCTCTTGCACA | - |  |
| 2 TBEV R1 |  | 1231 | CYGCAACCTTGGTRTCCGA | - |  |
|  |  |  |  |  |  |
| 3 TBEV F |  | 1128 | CACGYGAGTACTGTCTGCAT | + | 506 |
| 3 TBEV R2 |  | 1611 | AGCCAGATCATTGAACCAGTCAC | - |  |
| 3 TBEV R1 |  | 1670 | TTCAACCAGTCGCTCAGCATT | - |  |
|  |  |  |  |  |  |
| 4 TBEV F |  | 1619 | TTCAATGATCTGGCTCTACCG | + | 539 |
| 4 TBEV R2 |  | 2137 | ATGCTRCTCCCYTTYTGGAAC | - |  |
| 4 TBEV R1 |  | 2199 | GCGTGTTCTCCTATCACTGTCA | - |  |
|  |  |  |  |  |  |
| 5 TBEV F1 |  | 1964 | ACAAAACCYTGCAGGATYCCA | + | 799 |
| 5 TBEV F2 |  | 2029 | GCTGATAACACCAAACCCCACC | + |  |
| 5 TBEV R |  | 2806 | GCTCCARATCATTGAYTGKCCC | - |  |
|  |  |  |  |  |  |
| 6 TBEV F |  | 2690 | GAAGGRGAYGCAAACCTYAC | + | 753 |
| 6 TBEV R2 |  | 3422 | CCARCARTCAGTCCCCGTKCG | - |  |
| 6 TBEV R1 |  | 3478 | ACCATTGAYCGAACAAGACCC | - |  |
|  |  |  |  |  |  |
| 7 TBEV F1 |  | 3131 | GCCAGYCACACCATCGAC | + | 809 |
| 7 TBEV F2 |  | 3172 | GCTTTTCCTTCCAGCYAGCCT | + |  |
| 7 TBEV R |  | 3962 | CCATCARCAGGAGTCCGAC | - |  |
|  |  |  |  |  |  |
| 8 TBEV F |  | 3719 | CCWGAGATHGTGGCCCTGAC | + | 745 |
| 8 TBEV R2 |  | 4443 | CTTTCTCAAGCTCCGTCAGGT | - |  |
| 8 TBEV R1 |  | 4483 | GCAAGCCCAGCAAGYARCCAA | - |  |
|  |  |  |  |  |  |
| 9 TBEV F1 |  | 4253 | ATGCGYCACACKTCCCAGGAG | + | 710 |
| 9 TBEV F2 |  | 4287 | TCGCAGTRGCTTCATTCCTYC | + |  |
| 9 TBEV R |  | 4976 | CCYTTGGCTARGTCKATCGG | - |  |
|  |  |  |  |  |  |
| 10 TBEV F |  | 4877 | GTGCAGGTYCATGCYTTCCC | + | 891 |
| 10 TBEV R2 |  | 5746 | GCTGTTCARRCAAATCACGCTT | - |  |
| 10 TBEV R1 |  | 5812 | TCGGTCGTCACCACAAARTCA | - |  |
|  |  |  |  |  |  |
| 11 TBEV F |  | 5680 | CGCYTGGTTYGTTCCATCGATT | + | 906 |
| 11 TBEV R2 |  | 6565 | CTCTCYGCCATCCTCATTGCC | - |  |
| 11 TBEV R1 |  | 6597 | CCACCATCGTGAGAAAWGCCT | - |  |
|  |  |  |  |  |  |
| 12 TBEV F1 |  | 6349 | AAAYGGGGCTGAAAGRACGYTKA | + | 941 |
| 12 TBEV F2 |  | 6398 | TTCAGAGAAGGACGTGACATCA | + |  |
| 12 TBEV R |  | 7319 | TTGCGTACCATTGCCGAGAA | - |  |
|  |  |  |  |  |  |
| 13 TBEV F |  | 7239 | TTGGGYTRGCTGCMTTTCAYT | + | 818 |
| 13 TBEV R2 |  | 8036 | GCTGAASACATCCATTCCKGC | - |  |
| 13 TBEV R1 |  | 8094 | TCCGGGCTGCTTTCTCCRA | - |  |
|  |  |  |  |  |  |
| 14 TBEV F1 |  | 7852 | CGYGGCTATGCAACYCT | + | 935 |
| 14 TBEV F2 |  | 7913 | TGGTCCTACTATGCGGCCTC | + |  |
| 14 TBEV R |  | 8827 | TCCTCTTTGCTGCACATTCGT | - |  |
|  |  |  |  |  |  |
| 15 TBEV F |  | 8766 | CAAAGGTCATCATGAGRRCGGT | + | 838 |
| 15 TBEV R2 |  | 9584 | TCTTTCARCCAKCGTTCCAC | - |  |
| 15 TBEV R1 |  | 9620 | ACCACTGACGAGCATTCTTCC | - |  |
|  |  |  |  |  |  |
| 16 TBEV F |  | 9389 | GTGAAAGTTGCCAGGCCYTC | + | 782 |
| 16 TBEV R2 |  | 10149 | AGATACGGAATGTCTCTCCACT | - |  |
| 16 TBEV R1 |  | 10226 | TGTTCTTGGCCCAYTCRGC | - |  |
|  |  |  |  |  |  |
| 17 TBEV F |  | 9952 | CTATTTCCATCGGCGYGACYTG | + | 620 |
| 17 TBEV R2 |  | 10551 | CTCAGCCTCTTYCCYTTRCTT | - |  |
| 17 TBEV R1 |  | 10668 | GACCATTTCCCCGTCGCTCT | - |  |
|  |  |  |  |  |  |
| 18 TBEV F |  | 10314 | TGGACCGACATGACTTGCAYT | + | 514 |
| 18 TBEV R2 |  | 10808 | GACCATTTTCCCGTCGCCAC | - |  |
| 18 TBEV R1 |  | 10834 | AGTTTTAYAGAGTGCCCTACGTC | - |  |
|  |  |  |  |  |  |
| 19 TBEV F1 |  | 10587 | ATGACCAGACCGTTYTTACTGC | + | 452 |
| 19 TBEV F2 |  | 10629 | GAATGCGAGGCCACAKACCA | + |  |
| 19 TBEV R |  | 11063 | AGGKCCTCATCACCTCCT | - |  |

**Table S2.** **The primers used to detect tick-borne pathogens prevalent in the Daxing'an Mountains.**

| **Tick-borne pathogens** | **Target genes** | **Detection methods** | **Primer name** | **Sequence (5′→3′)** |
| --- | --- | --- | --- | --- |
| FE-TBEV | E | Nested PCR | TBEV-F1 | GGGAYTTTGTTACTGGCACTC |
|  |  |  | TBEV-R1 | TCAACRCCGCTGGCTACTC |
|  |  |  | TBEV-F2 | ATGGATGTGTGGCTTGACTC |
|  |  |  | TBEV-R2 | GAAACCGTGARGGATGCTGT |
| Beiji nairovirus | RdRP | SYBR RT-qPCR | BJ-F | TCACCAGTTCCTACTACG |
|  |  |  | BJ-R | GGACAACCTCAACATTCA |
| Songling virus | NP | SYBR RT-qPCR | SGLV-F | ATGGCACCTGTGTATGAG |
|  |  |  | SGLV-R | AGGCTTTCGTACTCCTTG |
| Alongshan virus | NSP2 | TaqMan RT-qPCR | ALSV-F | GGCTAAACACATCAAACA |
|  |  |  | ALSV-R | GCATCCAGGTCATAGTTA |
|  |  |  | ALSV-P | FAM-CCTTACACCACCATCGTGCTAAGC-BHQ1 |
| Wetland virus | RdRP | TaqMan RT-qPCR | WELV-L-F | TCAGATGATTATGCAAAGTGTATAGT |
|  |  |  | WELV-L-R | ACCGAGCTGTTTATCAGACCA |
|  |  |  | WELV-L-P | FAM-CAACGCTGCTGTCAAATGAAAGATAG-BHQ1 |
| Yezo virus | RdRP | TaqMan RT-qPCR | YEZV-F | GTCTGGGATCAAAGTCAG |
|  |  |  | YEZV-R | AGCACTTCATATTCTCCTTC |
|  |  |  | YEZV-P | FAM-AGAGTAAGGCACACCAGCATCAAC-BHQ1 |
| Anaplasma spp. | 16S rRNA | Semi-nested PCR | Eh-out1 | TTGAGAGTTTGATCCTGGCTCAGAACG |
|  |  |  | 3-17 | WAAGGTGGTAATCCAGC |
|  |  |  | Eh-out2 | CACCTCTACACTAGGAATTCCGCTATC |
| Babesia spp. | 18S rRNA | Nested PCR | BJ1-F1 | GTCTTGTAATTGGAATGATGG |
|  |  |  | BL-R1 | GAATAATTCACCGGATCACTCG |
|  |  |  | BJ1-F2 | GTCTTGTAATTGGAATGATGG |
|  |  |  | BL-R2 | ATTAACCAGACAAATCACTC |
| Borrelia spp. | 18S rRNA | Nested PCR | 23S3 | CGACCTTCTTCGCCTTAAAGC |
|  |  |  | 23Sa | TAAGCTGACTAATACTAATTACCC |
|  |  |  | 23S5 | CTGCGAGTTCGCGGGAGA |
|  |  |  | 23S6 | TCCTAGGCATTCACCATA |
| Rickettsia spp. | *gltA* | Nested PCR | CS2d | ATGACCAATGAAAATAATAAT |
|  |  |  | CSEndr | CTTATACTCTCTATGTACA |
|  |  |  | RpCS877 | GGGGACCTGCTCACGGCGG |
|  |  |  | RpCS1258 | ATTGCAAAAAGTACAGTGAACA |

**Table S3. Reference tick-borne encephalitis virus genome sequences utilized for** **phylogenetic analysis.**

| **TBEV subtype** | **GenBank accession number** | **Strain** | **Source** | **Place of isolation** | **Year of isolation** |
| --- | --- | --- | --- | --- | --- |
| Siberian-Vasilchenko | KF826916 | Sakhalin 6-11 | mosquitoes pool | Russia | 2011 |
| Siberian-Vasilchenko | LC017692 | MGL-Selenge-13-12 | *Ixodes persulcatus* | Mongolia | 2012 |
| Siberian-Vasilchenko | LC017693 | MGL-Selenge-13-14 | *Ixodes persulcatus* | Mongolia | 2012 |
| Siberian-Vasilchenko | JN003208 | Cht-22 | Human | Russia | 2002 |
| Siberian-Vasilchenko | JN003207 | Cht-653 | Human | Russia | 1995 |
| Siberian-Vasilchenko | KC414090 | Zabaikalye11-99 | Human | Russia | 1999 |
| Siberian-Vasilchenko | KF826914 | Zabaikalye1-09 | *Ixodes persulcatus* | Russia | 2009 |
| Siberian-Vasilchenko | KC422663 | Zabaikalye68B-00 | *Clethrionomys rutilus* | Russia | 2000 |
| Siberian-Vasilchenko | JN003206 | Aina | Human | Russia | 1963 |
| Siberian-Vasilchenko | JN003209 | Irkutsk-12 | Human | Russia | 2010 |
| Siberian-Vasilchenko | MN115820 | 518-66 | Human | Russia | 1966 |
| Siberian-Vasilchenko | AF069066 | Vasilchenko |  | Russia | 1969 |
| Siberian-Vasilchenko | FJ968751 | Kolarovo-2008 | *Ixodes pavlovskyi* | Russia | 2008 |
| Siberian-Vasilchenko | KM019545 | Tomsk-PT122 | *Acrocephalus dumetorum* | Russia | 2006 |
| Siberian-Vasilchenko | KP345889 | Sib-XJ-X5 | *Ixodes scapularis* | China | 2014 |
| Siberian-Vasilchenko | KF823822 | Irkutsk BR 683-11 | *Ixodes persulcatus* | Russia | 2011 |
| Siberian-Vasilchenko | PP473572 | HL9 | *Ixodes persulcatus* | China | 2022 |
| Siberian-Zausaev | JQ429588 | MucAr M14/10 | *Ixodes persulcatus* | Mongolia | 2010 |
| Siberian-Zausaev | KT321430 | Konst-14 | Human | Russia | 2014 |
| Siberian-Zausaev | MH645612 | 3869-03 | Human | Russia | 2003 |
| Siberian-Zausaev | MH645613 | Baikal-3 | *Ixodes persulcatus* | Russia | 1986 |
| Siberian-Zausaev | MH645614 | HimDym-6 | *Ixodes persulcatus* | Russia | 1986 |
| Siberian-Zausaev | MH645615 | Lukovka-3 | *Ixodes persulcatus* | Russia | 1986 |
| Siberian-Zausaev | KT224353 | LEIV-10133Al | *Ixodes persulcatus* | Russia | 1984 |
| Siberian-Zausaev | KJ701416 | Lesopark-11 | *Ixodes persulcatus* | Russia | 1986 |
| Siberian-Zausaev | KP644245 | C11-13 | Human | Russia | 2013 |
| Siberian-Zausaev | MH645619 | TBEV-2922 | *Ixodes pavlovskyi* | Russia | 2012 |
| Siberian-Zausaev | MH645618 | TBEV-2836 | *Ixodes pavlovskyi* | Russia | 2012 |
| Siberian-Zausaev | ON675587 | Novososedovo1 | *Ixodes persulcatus* | Russia | 2012 |
| Siberian-Zausaev | AF527415 | Zausaev | Human | Russia | 1985 |
| Siberian-Zausaev | MN114635 | TSA-18 | Human | Russia | 2018 |
| Siberian-Zausaev | LC017691 | IR99-22f7 | *Ixodes persulcatus* | Russia | 1999 |
| Siberian-Zausaev | MH645617 | TBEV-370 | *Ixodes persulcatus* | Russia | 2014 |
| Siberian-Zausaev | PP473572 | HL10 | *Ixodes persulcatus* | China | 2022 |
| Siberian-Zausaev | PP473573 | HL11 | *Ixodes persulcatus* | China | 2022 |
| Siberian-Baltic | KT224352 | LEIV-13652Ar | *Myodes glareolus* | Russia | 1984 |
| Siberian-Baltic | GU183382 | Latvia-1-96 | Mouse | Latvia | 1996 |
| Siberian-Baltic | DQ486861 | EK-328 |  | Estonia | 1972 |
| Siberian-Baltic | GU183384 | EST54 | Mouse | Estonia | 2000 |
| Siberian-Bosnia | KJ626343 | Buzuuchuk | *Ixodes persulcatus* | Kyrgyzstan | 1986 |
| Siberian-Bosnia | MH645616 | Bosnia-3 | *Ixodes ricinus* | Bosnia and Herzegovina | 2000 |
| Siberian-Obskaya | MF774565 | TBEV-2871 | *Ixodes pavlovskyi* | Russia | 2012 |
| Far-Eastern | JQ650523 | Senzhang | Human | China | 1953 |
| Far-Eastern | JQ650522 | MDJ01 | Human | China | 2001 |
| Far-Eastern | JX534167 | Xinjiang-01 | *Ixodes persulcatus* | China | 2012 |
| Far-Eastern | MN615728 | DXAL-T83 | *Ixodes persulcatus* | China | 2016 |
| Far-Eastern | MN615727 | HLB-T74 | *Ixodes persulcatus* | China | 2016 |
| Far-Eastern | ON408071 | NE-TH3 | *Ixodes persulcatus* | China | 2021 |
| Far-Eastern | LC440460 | Nanporo-18-44 | Ixodes tick | Japan | 2018 |
| Far-Eastern | JN003205 | Irkutsk-1861 | Human | Russia | 2008 |
| Baikalian | EF469662 | 886-84 | *Clethrionomys rufocanus* | Russia | 1984 |
| Himalaya | MG599476 | Himalaya-1 | *Marmota himalayana* | China | 2013 |
| Himalaya | MG599477 | Himalaya-2 | *Marmota himalayana* | China | 2013 |
| European | AM600965 | K23 |  | Germany | 1975 |
| European | U27495 | Neudoerfl | *Ixodes ricinus* | Austria | 1971 |
| European | U39292 | Hypr | Human | Czechoslovakia | 1953 |

**Table S4.** **Reference Sib-TBEV sequences utilized for Bayesian phylodynamic analysis.**

| **GenBank accession number** | **Strain** | **Place of isolation** | **Year of isolation** |
| --- | --- | --- | --- |
| MH681129 | 101-91 | Buryat Republic, Russia | 1991 |
| MH681131 | 108-80 | Buryat Republic, Russia | 1980 |
| MH681132 | 112-91 | Buryat Republic, Russia | 1991 |
| MH681134 | 122-80 | Buryat Republic, Russia | 1980 |
| MH681135 | 133-91 | Buryat Republic, Russia | 1991 |
| MH681138 | 143-87 | Buryat Republic, Russia | 1987 |
| MH681140 | 219-80 | Buryat Republic, Russia | 1980 |
| MH681142 | 238-88 | Buryat Republic, Russia | 1988 |
| MH681115 | 493-89 | Buryat Republic, Russia | 1989 |
| MH681120 | 757-90 | Zabaykalsky Krai, Russia | 1990 |
| MH681121 | 761-90 | Zabaykalsky Krai, Russia | 1990 |
| MH681122 | 769-90 | Zabaykalsky Krai, Russia | 1990 |
| MH681123 | 820-90 | Zabaykalsky Krai, Russia | 1990 |
| MH681124 | 84-91 | Buryat Republic, Russia | 1991 |
| MH681125 | 86-91 | Buryat Republic, Russia | 1991 |
| MH681126 | 878-90 | Buryat Republic, Russia | 1990 |
| MH681127 | 896-90 | Buryat Republic, Russia | 1990 |
| MH681128 | 97-91 | Buryat Republic, Russia | 1991 |
| MH681130 | 103-79 | Irkutsk region, Russia | 1979 |
| MH681133 | 117-91 | Irkutsk region, Russia | 1991 |
| MH681136 | 139-88 | Irkutsk region, Russia | 1988 |
| MH681137 | 141-88 | Irkutsk region, Russia | 1988 |
| MH681139 | 179-91 | Irkutsk region, Russia | 1991 |
| MH681141 | 236-91 | Irkutsk region, Russia | 1991 |
| MH681143 | 291-88 | Irkutsk region, Russia | 1988 |
| MH645612 | 3869-03 | Irkutsk region, Russia | 2003 |
| MH681116 | 56-87 | Irkutsk region, Russia | 1987 |
| MH681117 | 584-90 | Irkutsk region, Russia | 1990 |
| MH681118 | 59-87 | Irkutsk region, Russia | 1987 |
| MH681119 | 69-91 | Irkutsk region, Russia | 1991 |
| MH645613 | Baikal-3 | Irkutsk region, Russia | 1986 |
| MH645614 | HimDym-6 | Irkutsk region, Russia | 1986 |
| MH645615 | Lukovka-3 | Irkutsk region, Russia | 1986 |
| MG598840 | TBEV-154 | Novosibirsk region, Russia | 2013 |
| MG598850 | TBEV-186BB | Novosibirsk region, Russia | 2014 |
| MG598817 | TBEV-188-I-BB | Novosibirsk region, Russia | 2014 |
| MG598841 | TBEV-215 | Novosibirsk region, Russia | 2013 |
| MG598842 | TBEV-228 | Novosibirsk region, Russia | 2013 |
| MG598843 | TBEV-245 | Novosibirsk region, Russia | 2013 |
| MG598847 | TBEV-259-I-BK | Novosibirsk region, Russia | 2014 |
| MG598819 | TBEV-2730 | Novosibirsk region, Russia | 2011 |
| MG598820 | TBEV-2731 | Novosibirsk region, Russia | 2011 |
| MG598821 | TBEV-2736 | Novosibirsk region, Russia | 2011 |
| MG598822 | TBEV-2745 | Novosibirsk region, Russia | 2011 |
| MG598823 | TBEV-2746 | Novosibirsk region, Russia | 2011 |
| MG598824 | TBEV-2748 | Novosibirsk region, Russia | 2011 |
| MG598825 | TBEV-2779 | Novosibirsk region, Russia | 2011 |
| MG598826 | TBEV-2780 | Novosibirsk region, Russia | 2011 |
| MG598827 | TBEV-2781 | Novosibirsk region, Russia | 2011 |
| MG598828 | TBEV-2830 | Novosibirsk region, Russia | 2012 |
| MH645618 | TBEV-2836 | Novosibirsk region, Russia | 2012 |
| MG598830 | TBEV-2861 | Novosibirsk region, Russia | 2012 |
| MG598831 | TBEV-2869 | Novosibirsk region, Russia | 2012 |
| MF774565 | TBEV-2871 | Novosibirsk region, Russia | 2012 |
| MG598832 | TBEV-2874 | Novosibirsk region, Russia | 2012 |
| MG598838 | TBEV-29 | Novosibirsk region, Russia | 2013 |
| MG598833 | TBEV-2914 | Novosibirsk region, Russia | 2012 |
| MG598834 | TBEV-2917 | Novosibirsk region, Russia | 2012 |
| MH645619 | TBEV-2922 | Novosibirsk region, Russia | 2012 |
| MG598836 | TBEV-2926 | Novosibirsk region, Russia | 2012 |
| MG598837 | TBEV-2928 | Novosibirsk region, Russia | 2012 |
| MG598845 | TBEV-340 | Novosibirsk region, Russia | 2013 |
| MG598849 | TBEV-356ICR | Novosibirsk region, Russia | 2014 |
| MG598848 | TBEV-359BB | Novosibirsk region, Russia | 2014 |
| MH645617 | TBEV-370 | Novosibirsk region, Russia | 2014 |
| MG598846 | TBEV-386 | Novosibirsk region, Russia | 2013 |
| MG598839 | TBEV-72 | Novosibirsk region, Russia | 2013 |
| MH645616 | Bosnia-3 | Bosnia | 2000 |
| MH681113 | Bosnia-9 | Bosnia | 2000 |
| MH681114 | Bosnia-12 | Bosnia | 2000 |
| MH681107 | Crimea-2 | Crimean Peninsula | 1989 |
| MH681108 | Crimea-3 | Crimean Peninsula | 1989 |
| MH681109 | Crimea-4 | Crimean Peninsula | 1989 |
| MH681110 | Crimea-7 | Crimean Peninsula | 1989 |
| MH681111 | Crimea-8 | Crimean Peninsula | 1989 |
| MH681112 | Crimea-10 | Crimean Peninsula | 1989 |
| GU183382 | Latvia-1-96 | Latvia | 1996 |
| GU183384 | Est54 | Estonia | 2000 |
| DQ486861 | EK-328 | Estonia | 1972 |
| KU052689 | Karl08-T3522 | Karelia, Russia | 2008 |
| KJ744033 | Alma-Arasan LEIV-Kaz1380 | Kazakhstan | 1977 |
| KT224352 | LEIV-13652Ar | Arkhangelsk region, Russia | 1984 |
| EU444078 | Yar 114 | Yaroslavl Oblast, Russia | 2001 |
| EU444079 | Yar 46-2 | Yaroslavl Oblast, Russia | 2001 |
| EU444080 | Yar 48 | Yaroslavl Oblast, Russia | 2000 |
| EU444077 | Yar 71 | Yaroslavl Oblast, Russia | 1999 |
| GU125721 | YuK 4/13 | Kemerovo region, Russia | 1969 |
| KP644245 | C11-13 | Novosibirsk region, Russia | 2013 |
| JQ693478 | truncated | Novosibirsk region, Russia | 2010 |
| KJ701416 | Lesopark-11 | Novosibirsk region, Russia | 1986 |
| AF069066 | Vasilchenko | Novosibirsk region, Russia | 1969 |
| FJ968751 | Kolarovo-2008 | Tomsk region, Russia | 2008 |
| KM019545 | Tomsk-PT122 | Tomsk region, Russia | 2006 |
| AF527415 | Zausaev | Tomsk region, Russia | 1985 |
| KT224353 | LEIV-10133Al | Altai, Russia | 1984 |
| KJ626343 | Buzuuchuk | Kyrgyzstan | 1986 |
| JN003206 | Aina | Irkutsk region, Russia | 1963 |
| JN003208 | Cht-22 | Zabaykalsky Krai, Russia | 2002 |
| JN003207 | Cht-653 | Zabaykalsky Krai, Russia | 1995 |
| AB049348 | IR99-1m1 | Irkutsk region, Russia | 1999 |
| AB049349 | IR99-1m4 | Irkutsk region, Russia | 1999 |
| LC017691 | IR99-22f7 | Irkutsk region, Russia | 1999 |
| AB049353 | IR99-2f13 | Irkutsk region, Russia | 1999 |
| AB049352 | IR99-2f7 | Irkutsk region, Russia | 1999 |
| AB049350 | IR99-2m3 | Irkutsk region, Russia | 1999 |
| AB049351 | IR99-2m7 | Irkutsk region, Russia | 1999 |
| KF823822 | Irkutsk BR 683-11 | Irkutsk region, Russia | 2011 |
| JN003209 | Irkutsk-12 | Irkutsk region, Russia | 2010 |
| KT321430 | Konst-14 | Irkutsk region, Russia | 2014 |
| KU052690 | TV08-T2546 | Tyva Republic, Russia | 2008 |
| KF826914 | Zabaikalye 1-09 | Zabaykalsky Krai, Russia | 2009 |
| KC414090 | Zabaikalye 11-99 | Zabaykalsky Krai, Russia | 1999 |
| KC422663 | Zabaikalye 68B-00 | Zabaykalsky Krai, Russia | 2000 |
| HM133640 | 92M | Mongolia | 2004 |
| LC017692 | MGL-Selenge-13-12 | Selenge aimag, Mongolia | 2013 |
| LC017694 | MGL-Selenge-13-13 | Selenge aimag, Mongolia | 2013 |
| LC017693 | MGL-Selenge-13-14 | Selenge aimag, Mongolia | 2013 |
| LC017695 | MGL-Selenge-13-15 | Selenge aimag, Mongolia | 2013 |
| LC017696 | MGL-Selenge-13-18 | Selenge aimag, Mongolia | 2013 |
| LC017697 | MGL-Selenge-13-19 | Selenge aimag, Mongolia | 2013 |
| LC017698 | MGL-Selenge-13-21 | Selenge aimag, Mongolia | 2013 |
| KP666095 | MNG 940M | Mongolia | 2002 |
| KP666093 | MNG D13-15 | Mongolia | 2013 |
| KP666094 | MNG D13-37 | Mongolia | 2013 |
| JQ429588 | MucAr M14/10 | Mongolia | 2010 |
| KP345889 | Sib-XJ-X5 | Xinjiang, China | 2014 |
| KF826916 | Sakhalin 6-11 | Sakhalin island, Russia | 2011 |
| PP473573 | HL11 | China | 2022 |
| PP473572 | HL10 | China | 2022 |
| PP473571 | HL9 | China | 2022 |
| PP942934 | Tomsk 4-2023 | Tomsk Region, Russia | 2023 |
| PP942933 | Tomsk 3-2023 | Tomsk Region, Russia | 2023 |
| PP942932 | Tomsk 2-2023 | Tomsk Region, Russia | 2023 |
| PP942931 | Tomsk 1-2023 | Tomsk Region, Russia | 2023 |
| OP902894 | C11/13(mouse) | Novosibirsk, Russia | 2022 |
| OP902895 | C11/13-8m | Novosibirsk, Russia | 2022 |
| OQ565596 | C11-13/1p | Novosibirsk, Russia | 2013 |
| MN520113 | 562 | Eastern Siberia, Russia | 1960 |
| MN520114 | 253 | Eastern Siberia, Russia | 1963 |
| MT670184 | 559-66 | Eastern Siberia, Russia | 1966 |
| MN115818 | 41-65 | Irkutsk region, Russia | 1965 |
| MG589939 | Kuutsalo 2 Human Cerebellum Finland-2015 | Kuutsalo Island, Kotka archipelago, Finland | 2015 |
| MG589940 | Kotka-18 Ixodes ricinus Finland-2011 | Finland | 2011 |
| PQ014452 | Zababurikha 5-24 | Novosibirsk region, Koltsovo, Russia | 2024 |
| MN542364 | Rus/Ix persulcatus/Karelia/2/2018 | Gakugsa village, Karelia, Russia | 2018 |
| MN115820 | 518-66 | Irkutsk region, Russia | 1966 |
| MN520110 | 506 | Khiloksky/Petrovsk-Zabaykalsky districts, Russia | 1960 |
| MN520111 | 206 | Eastern Siberia, Russia | 1963 |
| MT670183 | 508-63 | Eastern Siberia, Russia | 1963 |
| PQ015165 | KY09 | Kyrgyzstan | 2009 |
| OR896869 | 23-Kyr-KDCA-26 | Kyrgyzstan | 2023 |
| MN115819 | 163-64 | Irkutsk region, Russia | 1964 |
| EF566817 | Z 6 | Kemerovo, Russia | 2005 |
| EF566818 | Z 7 | Kemerovo, Russia | 2005 |
| DQ451289 | Kokkola-26 | Finland | 2006 |
| DQ451296 | Kokkola-118 | Finland | 2006 |
| DQ451295 | Kokkola-102 | Finland | 2006 |
| FJ214130 | Kurgan-264-07 | Kurgan, Russia | 2007 |
| FJ214131 | Kurgan-273-07 | Kurgan, Russia | 2006 |
| FJ214149 | Kurgan-279-07 | Kurgan, Russia | 2007 |
| FJ214135 | Kemerovo-8-11-05 | Kemerovo, Russia | 2005 |
| GQ845423 | Bashkiria-351-09 | Bashkiria, Russia | 2009 |
| FJ214148 | Volkhov-2-43 | Volkhov, Leningrad Oblast, Russia | 1943 |
| GQ845422 | Chelyabinsk-357-09 | Ural, Chelyabinsk region, Russia | 2009 |
| KR633028 | Perm-Korz-86 | Perm, Russia | 1986 |
| KR633033 | Kirov-147-151-2012 | Kirov region, Russia | 2012 |
| KR633032 | Kirov-42-12 | Kirov region, Russia | 2012 |
| FJ214140 | Vologda-14-06 | Vologda Oblast, Russia | 2006 |
| FJ214141 | Vologda-15-06 | Vologda Oblast, Russia | 2006 |
| FJ214125 | Ekaterinburg-14-5-06 | Sverdlovsk region, Ural, Russia | 2006 |
| GQ845418 | Ekaterinburg-153-09 | Sverdlovsk region, Ural, Russia | 2009 |

**Table S5.** **Dates of clinical milestones and laboratory detection results of collected samples from seven patients infected with the Sib-TBEV.**

|  | HLB-ZH148 | HLB-ZG50 | HLB-ZH141 | HLB-H168 | HLB-H197 | HLB-H372 | ARS-H457 |
| --- | --- | --- | --- | --- | --- | --- | --- |
| Onset date | July 2, 2024 | June 16, 2023 | June 25, 2024 | June 11, 2024 | June 21, 2024 | June 14, 2024 | June 24, 2024 |
| Hospital visit date | July 5, 2024 | June 18, 2023 | July 1, 2024 | June 13, 2024 | June 24, 2024 | June 17, 2024 | June 27, 2024 |
| Sample collection date | July 6, 2024 | June 18, 2023 | July 3, 2024 | June 13, 2024 | June 24, 2024 | June 17, 2024 | June 27, 2024 |
| Collection days post onset | 4 | 2 | 8 | 2 | 3 | 3 | 3 |
| Sample type | Serum | Serum | Serum | Serum | Serum | Serum | Serum |
| RT-qPCR results | Positive | Positive | Positive | Positive | Positive | Positive | Positive |
| IgM/IgG results | Positive | Negative | Positive | Positive | Negative | Negative | Positive |
| CSF available | NA | NA | NA | NA | NA | NA | NA |

Abbreviations: CSF, Cerebrospinal fluid; NA, not available.

**Table S6. The information of the seven Sib-TBEV strains obtained from the patients in northeastern China.**

| **Subtype of TBEV** | **GenBank accession number** | **Strain** | **Source** | **Location** | **Year of patients** |
| --- | --- | --- | --- | --- | --- |
| Siberian-Vasilchenko | PQ790053 | HLB-ZH148 | Human | Hulunbuir, Inner Mongolia, China | 2024 |
| Siberian-Zausaev | PQ790051 | HLB-ZG50 | Human | Hulunbuir, Inner Mongolia, China | 2023 |
| Siberian-Zausaev | PQ790052 | HLB-ZH141 | Human | Hulunbuir, Inner Mongolia, China | 2024 |
| Siberian-Zausaev | PQ790054 | HLB-H168 | Human | Hulunbuir, Inner Mongolia, China | 2024 |
| Siberian-Zausaev | PQ790055 | HLB-H197 | Human | Hulunbuir, Inner Mongolia, China | 2024 |
| Siberian-Zausaev | PQ790056 | HLB-H372 | Human | Hulunbuir, Inner Mongolia, China | 2024 |
| Siberian-Zausaev | PQ790057 | ARS-H457 | Human | Hinggan League, Inner Mongolia, China | 2024 |
